# Maternal Age-Specific Rates for Trisomy 21 and Common Autosomal Trisomies in Fetuses from a Single Diagnostic Center in Thailand

**DOI:** 10.1371/journal.pone.0165859

**Published:** 2016-11-03

**Authors:** Kanoot Jaruthamsophon, Hutcha Sriplung, Chariyawan Charalsawadi, Pornprot Limprasert

**Affiliations:** 1 Human Genetics Unit, Department of Pathology, Faculty of Medicine, Prince of Songkla University, Hat Yai, Songkhla, Thailand; 2 Epidemiology Unit, Faculty of Medicine, Prince of Songkla University, Hat Yai, Songkhla, Thailand; University of Southern California, UNITED STATES

## Abstract

To provide maternal age-specific rates for trisomy 21 (T21) and common autosomal trisomies (including trisomies 21, 18 and 13) in fetuses. We retrospectively reviewed prenatal cytogenetic results obtained between 1990 and 2009 in Songklanagarind Hospital, a university teaching hospital, in southern Thailand. Maternal age-specific rates of T21 and common autosomal trisomies were established using different regression models, from which only the fittest models were used for the study. A total of 17,819 records were included in the statistical analysis. The fittest models for predicting rates of T21 and common autosomal trisomies were regression models with 2 parameters (Age and Age^2^). The rate of T21 ranged between 2.67 per 1,000 fetuses at the age of 34 and 71.06 per 1,000 at the age of 48. The rate of common autosomal trisomies ranged between 4.54 per 1,000 and 99.65 per 1,000 at the same ages. This report provides the first maternal age-specific rates for T21 and common autosomal trisomies fetuses in a Southeast Asian population and the largest case number of fetuses have ever been reported in Asians.

## Introduction

Many maternal age-specific rates for trisomy 21 (T21) in both fetuses and live births have been reported since the 1980s. In genetic counseling practice, these T21 rates in live births based on maternal age at date of delivery are widely applied in risk assessment and deciding whether to recommend prenatal screening, even though the rates used in counseling may not reflect the actual rates, as the live birth rates could be underestimating the risk as these rates are lower than the rates in fetuses at the amniocentesis date [1] and do not include the rates of all common autosomal trisomies (i.e. trisomies 21, 13 and 18). As the rates of T21 and common autosomal trisomies in fetuses have never been reported in any Southeast Asian population, this study was conducted to provide a maternal age-specific rate for T21 and common autosomal trisomies in southern Thailand for some baseline data for use in counseling practice.

Herein, we retrospectively analyzed cytogenetic findings from amniotic fluid cultures between 1990 and 2009 in a single diagnostic center, to calculate a referential maternal age-specific rate for T21 and common autosomal trisomies. We also compared our findings with previous reports in both fetuses and live births.

## Materials and Methods

We retrospectively reviewed the laboratory records of the Human Genetics Laboratory (Songklanagarind Hospital), Department of Pathology, Faculty of Medicine, Prince of Songkla University, which included amniotic fluid samples from 16 referral hospitals and clinics in southern Thailand. All records between 1990 and 2009 were reviewed for maternal age at the amniocentesis date (15^th^-20^th^ weeks of gestation), test indication and the result of amniotic fluid chromosome study. All chromosomal abnormalities were assigned following an International System for Human Cytogenetic Nomenclature (ISCN).

During the study period, 1990–1999, amniotic fluid cells were cultured by *in situ* method using a petri dish with a cover-slip. From 1999 to 2009, at least three primary cultures from two separate tubes were established in 24-well plates. Each culture was incubated at 37°C and 5% CO_2_ in different incubators. After enough cell colonies were obtained, metaphases chromosomes were harvested and prepared for standard G banding karyotype [2]. Karyotyping was done by light microscopy and photography until 1995, after which a digital analysis process was applied.

Only singleton pregnancy cases with the sole indication of advanced maternal (≥ 35 years of age at the time of delivery) were included for the statistical analysis. Herein, a portion of 34 year-old pregnant women whose age would be 35 at the estimated date of delivery were included. Mosaic T21, trisomy 18 (T18) and trisomy 13 (T13) cases were considered as trisomy cases in the analysis. Cases with Robertsonian T21 and T13 were not included in the trisomies cases in the analysis. We excluded the age group of more than 48 years from the statistical analysis due to the small number of these cases. As the numbers of cases with T18 and T13 were also small, we summarized the total number of trisomy cases and analyzed the maternal age-specific rate of common autosomal trisomies, instead of individual rates for T18 and T13. The statistical analysis was conducted with R software version 3.2.1. This research protocol was approved by the Research Ethics Committee, Faculty of Medicine, Prince of Songkla University (EC no. 58-136-05-1). Patient informed consent was waived with the approval of the Research Ethics Committee, Faculty of Medicine, Prince of Songkla University.

A logistic regression model and regression models with 2 and 3 parameters were calculated and plotted to choose the fittest model for predicting maternal age-specific rates (at the time of amniocentesis) for T21 and common autosomal trisomies (S1 and S2 Tables). The expected age-specific rate in each age group was calculated according to the selected model. All rates in this study were reported in the format of “number of cases per 1,000” as previously recommended [3].

The predicted rates of T21 and common autosomal trisomies were compared with previous reports in both fetuses and live births. For the T21 rate in live births, we included only reports published from 1995 and later in the comparison.

## Results

In the 20-year study period we analyzed a total of 19,818 amniotic fluid samples, of which 17,819 cases met the inclusion criteria for the study. There were 170, 52 and 16 cases with T21, T18 and T13, respectively (Table 1), and 2, 5 and 1 cases with mosaic T21, T18 and T13, respectively.

**Table 1:** Number of cases eligible for statistical analysis in each age group. Observed numbers of cases with common autosomal trisomies (trisomies 21, 18 and 13) and predicted maternal age-specific rates in each age group (at the time of amniocentesis).

Age at the time of amnio-centesis	Total cases	Observed abnormal cases				Predicted rates (per 1,000 fetuses)	
		T21	T18	T13	Common autosomal trisomies	T21	Common autosomal trisomies
34	621	2	0	0	2	2.67	4.54
35	2,781	8	6	0	14	2.93	4.61
36	3,206	12	3	1	16	3.90	5.71
37	2,698	17	4	4	25	5.58	7.84
38	2,487	23	6	1	30	7.98	11.01
39	1,900	15	8	2	25	11.09	15.22
40	1,533	19	8	2	29	14.91	20.46
41	1,022	19	4	0	23	19.44	26.73
42	723	25	5	1	31	24.69	34.04
43	442	14	4	3	21	30.65	42.39
44	214	8	1	0	9	37.32	51.77
45	113	5	2	1	8	44.70	62.19
46	50	1	0	1	2	52.80	73.64
47	15	1	0	0	1	61.61	86.14
48	14	1	1	0	2	71.14	99.66
**Total**	**17,819**	**170**	**52**	**16**	**238**		

T21: trisomy 21, T18: trisomy 18, T13: trisomy 13.

The fittest model of the maternal age-specific rate for T21 was a regression model of 2 parameters, *Rate* = 0.4181118 − (0.0243375 ∙ *Age*) + (0.0003564 ∙ *Age*^2^) (S1 and S3 Tables, S1 Fig). The fittest model of the maternal age-specific rate of common autosomal trisomies was also a regression model with 2 parameters, *Rate* = 0.6182885 − (0.0356498 ∙ *Age*) + (0.0005176 ∙ *Age*^2^) (S2 and S3 Tables, S2 Fig). The predicted rates of having a fetus with T21 were 2.67 per 1,000 fetuses at the age of 34 and 71.14 per 1,000 fetuses at the age of 48. The maternal age-specific rates for common autosomal trisomies at the ages of 34 and 48 were 4.54 and 99.66 per 1,000 fetuses, respectively (Table 1).

We compared our findings with 6 reported studies of T21 in fetuses [1,4–8] and 6 other studies in live births [9–14] (Table 2). Our maternal age-specific rates at the ages of 35 and 36 were lower than all other reported rates in fetuses, except the rate in one study which reported the lowest rate [6]. The rates at the maternal ages of 37 and above varied among the studies. We also found that almost all rates in live births were lower than the rates in fetuses. For example, the rates of T21 in fetuses at the age of 40 ranged from 8.98 to 17.54 per 1,000, while the rates in live births ranged from 7.30 to 11.77 per 1,000.

**Table 2:** Comparison of maternal age-specific rates of trisomy 21 per 1,000 fetuses and live births in various age groups from various studies.

Maternal age	Fetus rate at the time of amniocentesis							Live births rate at the time of delivery					
	This study	Hook 1983 [4]	Ferguson-Smith 1984 [5]	Yaegashi 1998 [6]	Snijders 1999 [1]^a^	Park 2010 [7]	Kim 2013 [8]^b^	Hecht and Hook 1996 [9]	Bray 1998 [10]	Huether 1998 [11]	Sheu 1998 [12]	Morris 2002 [13]	Metkeni 2005 [14]
34	2.67	3.10	N/A	N/A	2.86	N/A	N/A	2.27	2.40	2.39	3.17	2.20	1.50
35	2.93	4.00	3.80	2.62	3.57	3.68	7.78	2.81	2.97	2.94	4.00	2.84	2.30
36	3.90	5.20	4.90	3.37	4.55	4.59	9.15	3.56	3.72	3.68	4.83	3.76	2.60
37	5.58	6.70	6.30	4.31	5.85	5.71	10.77	4.60	4.73	4.68	5.46	5.03	3.90
38	7.98	8.70	8.10	5.50	7.63	7.09	12.67	6.03	6.06	6.03	5.71	6.76	4.60
39	11.09	11.20	10.40	7.03	10.00	8.85	14.91	8.00	7.82	7.88	6.02	9.01	6.90
40	14.91	14.50	13.30	8.98	13.16	10.99	17.54	10.68	10.15	10.46	7.30	11.77	9.20
41	19.44	18.70	17.10	11.47	17.54	13.70	20.64	14.29	13.24	14.08	8.93	14.93	13.70
42	24.69	24.10	21.80	14.66	23.26	17.24	24.29	19.06	17.31	19.22	9.43	18.52	14.70
43	30.65	31.10	27.90	18.73	31.25	21.28	28.58	25.21	22.67	26.58	N/A	22.22	23.20
44	37.32	40.10	35.60	23.93	41.67	26.32	33.63	32.86	29.70	37.18	N/A	25.64	26.20
45	44.70	51.80	45.30	30.57	55.56	33.33	39.57	41.93	38.89	52.55	N/A	28.57	27.40
46	52.80	66.8	57.5	N/A	N/A	N/A	46.57	52.03	50.83	52.55	N/A	32.26	33.3
47	61.61	86.2	23.3	N/A	N/A	N/A	54.79	62.32	66.26	52.55	N/A	34.48	44.7
48	71.14	111.2	23.3	N/A	N/A	N/A	N/A	71.53	86.00	52.55	N/A	37.04	51.9
Sample size	17,819	N/A	52,965	5,484	57,614	2,032	5,055	1,615,142	N/A	N/A	7,232,689	6,008,450	4,139,205
Study location	Thailand	Mixed	Europe	Japan	US	Korea	Korea	Mixed	Mixed	South-west Ohio and Atlanta (White US)	Taiwan	England & Wales	Hungary

N/A: not available.

^a^At the gestational age of 16 weeks.

^b^Age at the time of delivery.

For the predicted maternal age-specific rate for common autosomal trisomies, we found 6 studies which reported the rates in one-year intervals, five studies on rates in fetuses [4–8] and one rate in live births [15]. The maternal age-specific rates for common autosomal trisomies in fetuses ranged from 3.94 to 7.92 per 1,000 at the age of 35 and from 24.31 to 42.47 per 1,000 at the age of 42. The only rate in live births reported the lowest rate than other reports in fetuses (Table 3).

**Table 3:** Comparison of maternal age-specific rates for common autosomal trisomies (trisomies 21, 18 and 13) and aneuploidies per 1,000 fetuses and live births in various age groups from various studies.

Maternal age	Fetus rate at the time of amniocentesis						Live births rate at the time of delivery
	This study	Hook 1983 [4]	Ferguson-Smith 1984 [5]	Yaegashi 1998 [6]	Park 2010 [7]	Kim 2013 [8]^a^	Savva 2010 [15]
	T21, T13, T18	T21, T13, T18	T21, T13, T18	T21, T13, T18	All aneuploidies	T21, T13, T18	T21, T18, T13
34	4.54	4.30	N/A	N/A	N/A	N/A	1.83
35	4.61	5.50	4.50	3.94	7.92	11.14	2.22
36	5.71	7.10	5.90	5.13	10.06	13.15	2.81
37	7.84	8.90	7.70	6.64	12.79	15.54	3.64
38	11.01	11.50	10.00	8.6	16.26	18.35	4.85
39	15.22	14.70	13.20	11.15	20.67	21.68	6.53
40	20.46	18.80	17.20	14.45	26.28	25.62	8.78
41	26.73	24.00	22.50	18.73	33.41	30.28	11.67
42	34.04	30.60	29.40	24.31	42.47	35.80	15.18
43	42.39	39.20	36.00	31.55	53.99	42.34	19.18
44	51.77	50.30	40.90	40.96	68.63	50.08	23.47
45	62.19	64.40	50.60	53.19	87.25	59.26	27.79
46	73.64	82.5	62.80	N/A	N/A	70.13	31.90
47	86.14	105.8	28.6	N/A	N/A	83.04	35.65
48	99.66	135.6	28.6	N/A	N/A	N/A	38.91
Sample size	17,819	N/A	52,965	5,484	2,032	5,055	4.5 million
Study location	Thailand	Mixed	Europe	Japan	Korea	Korea	Mixed

T21: trisomy 21, T18: trisomy 18, T13: trisomy 13, N/A: not available.

^a^Age at the time of delivery.

## Discussion

Our study is the first report from Thailand and the largest to date from Asia providing maternal age-specific rates of the likelihood of carrying a fetus with T21 and common autosomal trisomies. For the analysis, we applied a regression model with 2 parameters, instead of the conventional logistic regression model, to adjust the predicted risk to match actual observed frequencies as closely as possible. Both models seemed to fit very well from the ages of 34 to 41, but at and after the age 42 the rates predicted by logistic regression increased steadily, to much higher levels than in the chosen regression model with 2 parameters, for both the T21 rate and the rates of common autosomal trisomies (S1 and S2 Tables, S1 and S2 Figs). As the T21 rate at the age of more than 45 was observed to stop increasing, presumably due to very early spontaneous abortion [16], the continual increment of the rate in the logistic regression model was thus contradictory. These findings may well be the result of the lower numbers of cases (N <1,000) in the 42-and-higher age groups (Table 1). However, even if this finding represented only a typical statistical variation, it does underline the importance of model choosing, which can affect risk assessment.

The reported rates of T21 in fetuses, especially those with a sample size of less than 10,000 cases, have varied much more among studies than rates in live births (Table 2). Because of the small sample sizes in fetal studies, the predicted rates could be affected by imprecision in measuring the maternal age-specific rates for T21. In contrast, all studies involving live births were based on sample sizes of more than 100,000 cases. The degree of variation and standardization of the reports based on live births were also much well-studied than in fetuses [10,17]. However, variations in T21 rates were still observed in extremely advanced age groups–among both fetuses and live births studies–in which case numbers per age group were relatively small.

In conclusion, this study provides the first maternal age-specific rates of fetal T21 in the Thai population, based on the largest fetal case numbers of maternal age-specific rates for T21 and common autosomal trisomies to date in an Asian study. Our data is an alternative information that could be applied to risk assessment in genetic counseling for prenatal screening and decision making in public health services.

## Supporting Information

S1 Fig Predicted maternal age-specific rates for trisomy 21 at ages 34–48 years. The dots represent observed frequencies of T21 at each age. The black lines represent the 95% confidence intervals of the observed frequencies in each age. The blue line represents the predicted maternal age-specific rate for T21 based on the logistic regression model. The pink line represents the predicted maternal age-specific rate for T21 based on the 2 parameters (Age and Age^2^) regression model.
(DOCX)

S2 Fig Predicted maternal age-specific rates for common autosomal trisomies at ages 34–48 years. The dots represent observed frequencies of common autosomal trisomies in each age. The black lines represent the 95% confidence intervals of the observed frequencies in each age. The blue line represents the predicted maternal age-specific rate for common autosomal trisomies based on the logistic regression model. The pink line represents the predicted maternal age-specific rate for common autosomal trisomies based on the 2 parameters (Age and Age^2^) regression model.
(DOCX)

S1 Table Predicted models for maternal age-specific risk for trisomy 21. The predicted models include a logistic regression model, regression models with 2 parameters and a regression model with 3 parameters. The chosen model was the regression model with 2 parameters (Age and Age^2^).
(DOCX)

S2 Table Predicted models for maternal age-specific risk for common autosomal trisomies. The predicted models include a logistic regression model, regression models with 2 parameters and a regression model with 3 parameters. The chosen model was the regression model with 2 parameters (Age and Age^2^).
(DOCX)

S3 Table A comparison between predicted rates of having a fetus with trisomy 21 and common autosomal trisomies at different ages. The predicted rates were calculated by the logistic regression model and the regression model with 2 parameters (Age and Age^2^).
(DOCX)

S4 Table Number of cases eligible for statistical analysis in each gestational age group. (DOCX)
