# Supplementary material for: Maternal Age-Specific Rates for Trisomy 21 and Common Autosomal Trisomies in Fetuses from a Single Diagnostic Center in Thailand
Source: PLoS One. 2016 Nov 3;11(11):e0165859. doi: 10.1371/journal.pone.0165859 (PMC5094691; doi:10.1371/journal.pone.0165859)
Supplement: S2 Fig — The dots represent observed frequencies of common autosomal trisomies in each age. The black lines represent the 95% confidence intervals of the observed frequencies in each age. The blue line represents the predicted maternal age-specific rate for common autosomal trisomies based on the logistic regression model. The pink line represents the predicted maternal age-specific rate for common autosomal trisomies based on the 2 parameters (Age and Age2) regression model. (DOCX) [file pone.0165859.s002.docx]

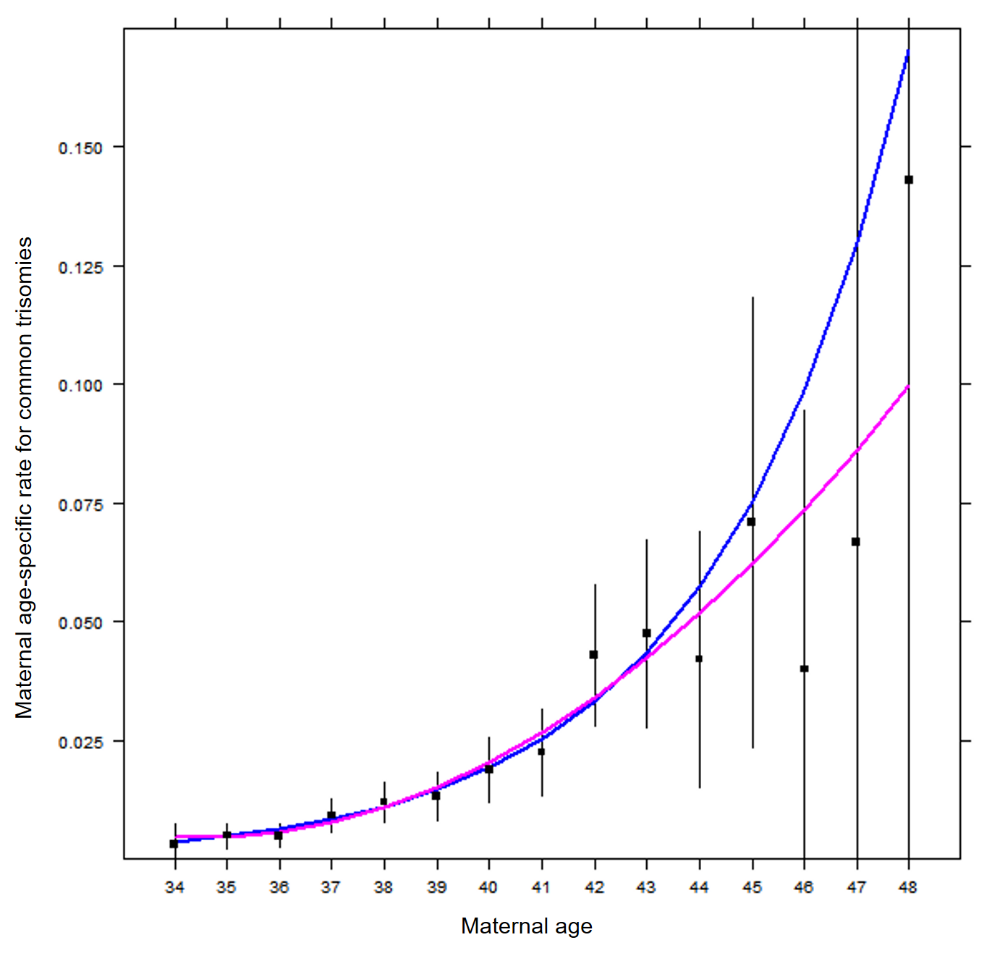


**S2 Fig. Predicted maternal age-specific rates for common autosomal trisomies at ages 34-48 years.** The dots represent observed frequencies of common autosomal trisomies in each age. The black lines represent the 95% confidence intervals of the observed frequencies in each age. The blue line represents the predicted maternal age-specific rate for common autosomal trisomies based on the logistic regression model. The pink line represents the predicted maternal age-specific rate for common autosomal trisomies based on the 2 parameters (Age and Age^2^) regression model.
