# Supplementary material for: Maternal Age-Specific Rates for Trisomy 21 and Common Autosomal Trisomies in Fetuses from a Single Diagnostic Center in Thailand
Source: PLoS One. 2016 Nov 3;11(11):e0165859. doi: 10.1371/journal.pone.0165859 (PMC5094691; doi:10.1371/journal.pone.0165859)
Supplement: S1 Table — The predicted models include a logistic regression model, regression models with 2 parameters and a regression model with 3 parameters. The chosen model was the regression model with 2 parameters (Age and Age2). (DOCX) [file pone.0165859.s003.docx]

**S1 Table. Predicted models for maternal age-specific risk for trisomy 21.** The predicted models include a logistic regression model, regression models with 2 parameters and a regression model with 3 parameters. The chosen model was the regression model with 2 parameters (Age and Age^2^)**.**

| **Model** | **Predictor parameter** | **Coefficient** | **z value/ t-value** | **Intercept** | **z value/ t-value** | **AIC** |
| --- | --- | --- | --- | --- | --- | --- |
| Logistic regression for trisomy 21 | Age | 0.27753 | z value 10.70 | -15.39332 | z value -14.87 | 1819.3 |
| Regression model with 2 parameters | Age  Age^2^ | -0.0243375  0.0003564 | t-value -2.832  t-value 3.217 | 0.4181118 | t-value 2.518 | -109.46 |
|  | Age  Age^3^ | -0.01022  2.988 x 10^-6^ | t-value -2.390  t-value 3.168 | 0.2326 | t-value 2.108 | -109.25 |
|  | Age^2^  Age^3^ | -2.569 x 10^-4^  5.131 x 10^-6^ | t-value -2.349  t-value 2.743 | 9.759 x 10^-2^ | t-value 1.778 | -109.08 |
| Regression model with 3 parameters | Age  Age^2^  Age^3^ | -0.1352  3.179 x 10^-3^  -2.386 x 10^-5^ | t-value -0.954  t-value 0.882  t-value -0.784 | 1.863 | t-value 1.006 | -108.27 |

AIC: Akaike information criterion
