# Supplementary material for: Maternal Age-Specific Rates for Trisomy 21 and Common Autosomal Trisomies in Fetuses from a Single Diagnostic Center in Thailand
Source: PLoS One. 2016 Nov 3;11(11):e0165859. doi: 10.1371/journal.pone.0165859 (PMC5094691; doi:10.1371/journal.pone.0165859)
Supplement: S2 Table — The predicted models include a logistic regression model, regression models with 2 parameters and a regression model with 3 parameters. The chosen model was the regression model with 2 parameters (Age and Age2). (DOCX) [file pone.0165859.s004.docx]

**S2 Table. Predicted models for maternal age-specific risk for common autosomal trisomies.** The predicted models include a logistic regression model, regression models with 2 parameters and a regression model with 3 parameters. The chosen model was the regression model with 2 parameters (Age and Age^2^).

| **Model** | **Predictor parameter** | **Coefficient** | **z value/ t-value** | **Intercept** | **z value/ t-value** | **AIC** |
| --- | --- | --- | --- | --- | --- | --- |
| Logistic regression for common autosomal trisomies | Age | 0.27249 | z value 12.31 | -14.84892 | z value -16.83 | 2391.6 |
| Regression model with 2 parameters | Age  Age^2^ | -0.0356498  0.0005176 | t-value -3.497  t-value 3.937 | 0.6182885 | t-value 3.138 | -104.33 |
|  | Age  Age^3^ | -1.531 x 10^-2^  4.373 x 10^-6^ | t-value -3.036  t-value 3.933 | 0.3528 | t-value 2.713 | -104.31 |
|  | Age^2^  Age^3^ | -3.886 x 10^-4^  7.648 x 10^-6^ | t-value -3.027  t-value 3.484 | 0.1527 | t-value 2.370 | -104.27 |
| Regression model with 3 parameters | Age  Age^2^  Age^3^ | -3.541 x 10^-2^  5.115 x 10^-4^  5.165 x 10^-8^ | t-value -0.205  t-value 0.116  t-value 0.001 | 0.6152 | t-value 0.273 | -102.33 |

AIC: Akaike information criterion
