# Supplementary material for: Maternal Age-Specific Rates for Trisomy 21 and Common Autosomal Trisomies in Fetuses from a Single Diagnostic Center in Thailand
Source: PLoS One. 2016 Nov 3;11(11):e0165859. doi: 10.1371/journal.pone.0165859 (PMC5094691; doi:10.1371/journal.pone.0165859)
Supplement: S3 Table — The predicted rates were calculated by the logistic regression model and the regression model with 2 parameters (Age and Age2). (DOCX) [file pone.0165859.s005.docx]

**S3 Table. A comparison between predicted rates of having a fetus with trisomy 21 and common autosomal trisomies at different ages.** The predicted rates were calculated by the logistic regression model and the regression model with 2 parameters (Age and Age^2^).

| **Age at the time of amnio-centesis** | **Trisomy 21** | | **Common autosomal trisomies** | |
| --- | --- | --- | --- | --- |
|  | **Logistic regression model** | **Chosen regression model with  2 parameters**  **(Age and Age^2^)** | **Logistic regression model** | **Chosen regression model with  2 parameters**  **(Age and Age^2^)** |
|  | **Rate**  **(cases per 1,000)** | **Rate**  **(cases per 1,000)** | **Rate**  **(cases per 1,000)** | **Rate**  **(cases per 1,000)** |
| 34 | 2.59 | 2.67 | 3.76 | 4.54 |
| 35 | 3.41 | 2.93 | 4.93 | 4.61 |
| 36 | 4.51 | 3.90 | 6.48 | 5.71 |
| 37 | 5.95 | 5.58 | 8.51 | 7.84 |
| 38 | 7.85 | 7.98 | 11.17 | 11.01 |
| 39 | 10.36 | 11.09 | 14.67 | 15.22 |
| 40 | 13.68 | 14.91 | 19.27 | 20.46 |
| 41 | 18.05 | 19.44 | 25.30 | 26.73 |
| 42 | 23.82 | 24.69 | 33.23 | 34.04 |
| 43 | 31.44 | 30.65 | 43.63 | 42.39 |
| 44 | 41.50 | 37.32 | 57.30 | 51.77 |
| 45 | 54.78 | 44.70 | 75.25 | 62.19 |
| 46 | 72.30 | 52.80 | 98.82 | 73.64 |
| 47 | 95.43 | 61.61 | 129.77 | 86.14 |
| 48 | 125.95 | 71.14 | 170.41 | 99.66 |
