# Supplementary material for: Maternal Age-Specific Rates for Trisomy 21 and Common Autosomal Trisomies in Fetuses from a Single Diagnostic Center in Thailand
Source: PLoS One. 2016 Nov 3;11(11):e0165859. doi: 10.1371/journal.pone.0165859 (PMC5094691; doi:10.1371/journal.pone.0165859)
Supplement: S4 Table — (DOCX) [file pone.0165859.s006.docx]

**S4 Table. Number of cases eligible for statistical analysis in each gestational age group.**

| **Gestational age at the time of amniocentesis (weeks)** | **Total cases** | **Percentage** |
| --- | --- | --- |
| 15 | 273 | 1.5 |
| 16 | 1,994 | 11.2 |
| 17 | 4,780 | 26.8 |
| 18 | 5,271 | 29.6 |
| 19 | 3,103 | 17.4 |
| 20 | 1,412 | 8.0 |
| Not available | 986 | 5.5 |
| **Total** | **17,819** | **100** |
